# Blood Genome-Wide Transcriptional Profiles of HER2 Negative Breast Cancers Patients

**DOI:** 10.1155/2016/3239167

**Published:** 2016-01-13

**Authors:** Ovidiu Balacescu, Loredana Balacescu, Oana Virtic, Simona Visan, Claudia Gherman, Flaviu Drigla, Laura Pop, Gabriela Bolba-Morar, Carmen Lisencu, Bogdan Fetica, Oana Tudoran, Ioana Berindan-Neagoe

**Affiliations:** ^1^Department of Functional Genomics and Experimental Pathology, The Oncology Institute “Prof. Dr. Ion Chiricuta”, 34-36 Republicii Street, 400015 Cluj-Napoca, Romania; ^2^Research Center for Functional Genomics, Biomedicine and Translational Medicine, University of Medicine and Pharmacy “I. Hatieganu”, 23 Marinescu Street, 400337 Cluj-Napoca, Romania; ^3^Department of Senology, The Oncology Institute “Prof. Dr. Ion Chiricuta”, 34-36 Republicii Street, 400015 Cluj-Napoca, Romania; ^4^Department of Imagistics, The Oncology Institute “Prof. Dr. Ion Chiricuta”, 34-36 Republicii Street, 400015 Cluj-Napoca, Romania; ^5^Department of Pathology, The Oncology Institute “Prof. Dr. Ion Chiricuta”, 34-36 Republicii Street, 400015 Cluj-Napoca, Romania

## Abstract

Tumors act systemically to sustain cancer progression, affecting the physiological processes in the host and triggering responses in the blood circulating cells. In this study, we explored blood transcriptional patterns of patients with two subtypes of HER2 negative breast cancers, with different prognosis and therapeutic outcome. Peripheral blood samples from seven healthy female donors and 29 women with breast cancer including 14 triple-negative breast cancers and 15 hormone-dependent breast cancers were evaluated by microarray. We also evaluated the stroma in primary tumors. Transcriptional analysis revealed distinct molecular signatures in the blood of HER2− breast cancer patients according to ER/PR status. Our data showed the implication of immune signaling in both breast cancer subtypes with an enrichment of these processes in the blood of TNBC patients. We observed a significant alteration of “chemokine signaling,” “IL-8 signaling,” and “communication between innate and adaptive immune cells” pathways in the blood of TNBC patients correlated with an increased inflammation and necrosis in their primary tumors. Overall, our data indicate that the presence of triple-negative breast cancer is associated with an enrichment of altered systemic immune-related pathways, suggesting that immunotherapy could possibly be synergistic to the chemotherapy, to improve the clinical outcome of these patients.

## 1. Introduction

Breast cancer, the most diagnosed malignancy in women [1], is a highly heterogeneous disease presenting a broad range of molecular, biological, and clinical characteristics. Despite the advances in molecular classification of breast cancer [2–5], identifying of clinically relevant subgroups is still based on the status of estrogen and progesterone receptor (ER and PR) and human epidermal growth factor receptor 2 (HER2) along with clinicopathological variables. Currently, breast cancer is categorized into three main therapeutic groups: ER-positive (ER+), HER2-positive (HER2+), and triple-negative breast cancer (TNBC/ER−PR−HER2−). ER+ tumors account for about 70% of breast cancer that respond well to endocrine therapy and have a good prognosis and survival (5-year survival rate of 85%) [6]. Among ER+ tumors, HER2 negativity is associated with a better prognosis when compared with HER2+ tumors. Overall, overexpression of HER2, identified in about 20% of breast cancer, is associated with a more aggressive phenotype but, however, survival of these patients has been dramatically improved by the development of drugs targeting this receptor (trastuzumab, lapatinib, and pertuzumab) [7]. Unlike the ER+ or HER2+ breast cancers, triple-negative tumors lack a validated targeted therapy, with conventional chemotherapy remaining the standard of care. As a result, TNBC subtype tends to have a poor clinical outcome and an increased risk of recurrence and distant metastasis. Therefore, there is a major concern regarding the identification of new therapeutic targets for this subtype and developing an effective targeted therapy for these patients.

Gene expression profiling of peripheral blood cells arises as a valuable tool to evaluate gene signatures related to solid tumors. The reason to use blood cells as “sensors” to characterize tissue tumors is based on the fact that blood circulating cells monitor the body's physiological status and modify their expression pattern in response to pathological changes. Previous studies on peripheral blood revealed specific signatures related to lymphomas and leukemia as well as inflammatory and autoimmune diseases [8–10]. Gene expression signatures in peripheral blood of breast cancer patients were associated with early detection of tumors [11, 12], predicting metastasis [13, 14], or treatment response to therapy [15]. However, the tumor-blood communication involves a large spectrum of signaling molecules and deciphering their role still represents a great challenge.

In line with this view, the overall aim of this study was to evaluate the mRNA-peripheral blood profile of two HER2− breast cancer subtypes, including hormone-dependent breast cancer (ER+PR+HER2−) and triple-negative breast cancer (TNBC/ER−PR−HER2−), known to have the best and the worst prognosis, respectively.

## 2. Materials and Methods

### 2.1. Blood Sample Collection and Processing

Twenty-nine female breast cancer patients were recruited for this study between August 2010 and September 2012 at The Oncology Institute “Prof. Dr. Ion Chiricuta,” Cluj-Napoca (IOCN), Romania. The study was approved by the ethical committees of the University of Medicine and Pharmacy “Iuliu Hatieganu,” Cluj-Napoca, Romania, and the IOCN, the coordinators of this study. All patients provided informed consent in accordance with the Declaration of Helsinki. The patients were included in the study if they met the following criteria: (a) were recently diagnosed with invasive breast cancer, (b) had negative HER2 status (HER2−) in the primary tumors, (c) did not present metastasis or secondary malignancies, and (d) were not treated prior to or during the collection of biological samples. The status of ER, PR and HER2 was assessed by immunohistochemistry and staging was done according to AJCC criteria by a certified pathologist (Table 1). Additionally, a group of 7 healthy women was considered as control (CTR).

From each subject, 4 mL of peripheral blood was collected in EDTA anticoagulant tubes. At the time of collection, none of the participants had fever or any acute diseases, followed anticoagulant therapy, or received chemotherapy, radiotherapy, or immunotherapy. Blood samples were collected between 8 a.m. and 12 p.m. for all subjects. They were immediately stored on ice and processed following a standardized protocol. Briefly, after plasma removal and RBC lysis, total RNA from nucleated blood cells was extracted using TriReagent (Ambion/Thermo Fisher, Waltham, MA, USA) and purified with the RNeasy Mini kit (Qiagen, Hilden, Germany) according to the manufacturer's protocols. The quality of purified RNA was assessed with a Bioanalyzer 2100 (Agilent Technologies, Santa Clara, CA, USA) and a Nanodrop-ND-1000 spectrophotometer (NanoDrop Technologies, Wilmington, DE, USA) was used to quantify the extracted RNAs.

RNA Integrity Number (RIN) was used to define the quality of RNAs. All extracted RNAs had RIN between 8 and 10 and were considered for further analysis. The RNAs were stored at −80°C until further processing for microarray.

### 2.2. Microarray Experiment

One hundred nanograms of total RNA was used for the synthesis of one-color microarray probes using the Agilent Low Input Quick Amp Labeling Kit according to Agilent's protocols. Before hybridization, microarray probes (cRNA-Cy3) were purified with the RNeasy Mini Kit (Qiagen, Hilden, Germany). All microarray probes with a minimum concentration of 1.65 *μ*g and specific activities of 6 pmol/*μ*L Cy3 were considered for hybridization. The cRNAs-Cy3 probes were hybridized for 17 hours at 65°C on human gene expression 4 × 44 k v2 microarray slides (G4845A) following the manufacturer's protocol (Agilent Technologies, Santa Clara, CA, USA). Slides were scanned with an Agilent G2565CA microarray scanner, and image processing was done using Feature Extraction Software v.11.0.

### 2.3. Microarray Data Analysis

The datasets containing array signal intensities were imported and analyzed in Gene Spring GX v.11.5. Quantile normalization was used to correct for interarrays global differences. Nonuniform, outlier, and saturated spots were filtered, and only sequences with acceptable flags in minimum 90% samples were retained for analysis. Differences in gene expression between the three studied groups (ER−PR−HER2−, ER+PR+HER2−, and CTR) were tested by using one-way ANOVA followed by Tukey's post hoc test. When two groups were considered (all breast cancer samples, named as BC, versus CTR) differences in gene expression were assessed by unpaired *t*-test. For all of the comparisons, *p* values were adjusted for multiple testing by the Benjamini-Hochberg FDR method. Genes were considered to be differentially expressed when their expression exceeded 1.5-fold between groups and the adjusted *p* value was less than 0.05. Differentially expressed gene profiles were further used to compute a supervised cluster based on the Euclidean distances and Ward algorithm.

### 2.4. Ingenuity Pathways Analysis (IPA)

The four lists of differentially expressed genes between groups, containing Agilent probe set IDs and fold changes, were uploaded into IPA (Ingenuity Systems, http://www.ingenuity.com/) and queried against a background specific model (Agilent Whole Human Genome Microarray 4 × 44 k v2). IPA Core Analysis function was used to examine which biological processes and pathways were affected by gene expression changes observed in our datasets and also to identify upstream regulators (UR) and their targets that could control these processes. The significance of the association between each dataset and functional categories or canonical pathways stored in Ingenuity Knowledge Base was tested by Fisher's exact test. The threshold of significance was set at 0.05. In order to predict significant URs, an overlap *p* value and an activation *z*-score were computed for each potential UR. The overlap *p* value was estimated by Fisher's exact test, indicating whether there is a significant overlap between the genes in our dataset and the genes known to be modulated by an UR. An overlap *p* value less than 0.01 was considered significant. Activation *z*-score was assigned based on the consistency between the expected effects (activation or inhibition) of an UR on each target gene and the observed changes in gene expression. Thus, UR was predicted to be in an “activated” state if *z*-score > 2; otherwise UR was predicted to be in an “inhibited” state (*z*-score < −2).

### 2.5. Quantitative Real-Time PCR (qRT-PCR)

Quantitative real-time PCR analysis was used to validate the microarray results. One hundred nanograms of total RNA for every sample was reverse-transcribed to cDNA using First Strand cDNA Synthesis Kit (Roche Applied Science, Penzberg, Germany). Two and a half microliters of 1 : 10 (v/v) diluted cDNA was amplified with 1 *μ*M of specific primers and 0.2 *μ*M of fluorescence probes in a final volume of 10 *μ*L using LightCycler Taqman Master Kit (Roche Applied Science, Penzberg, Germany). Roche Applied Science software was used to design the structure of primers and specific Universal Probe Library (UPL) probe for every gene as follows: PTGS2 (NM_000963.2): F-cttcacgcatcagtttttcaag, R-tcaccgtaaatatgatttaagtccac, UPL (#23); IL-8 (NM_000584.3): F-gagcactccataaggcacaaa, R-atggttccttccggtggt, UPL (#72); TREM1 (NM_018643.3): F-tctggactgtatcagtgtgtgatct, R-ccaggggtccctgaaaaa, UPL (#75); AREG (NM_001657.2): F-tgatcctcacagctgttgct, R-tccattctcttgtcgaagtttct, UPL (#73); RNA18S5 rRNA (NR_003286.2): F-gcaattattccccatgaacg, R-gggacttaatcaacgcacgc, UPL (#48); RPLP0 (NM-001002.3): F-gatgcccagggaagacag, R-tctgctcccacaatgaaacat, UPL (#85). Thermal cycling conditions were performed in a ViiA7 system (Applied Biosystems) and included a denaturation step at 95°C for 15 seconds followed by 40 cycles of amplifications consisting of an annealing step at 60°C for 20 seconds and extension at 72°C for 1 second. RPLP0 and RNA18S5 housekeeping genes were used to normalize the genes of interest, and their relative expression was calculated using ΔΔCt relative quantification method.

### 2.6. Stroma Evaluation

Hematoxylin and Eosin- (H&E-) stained slides on 5 *μ*m tissue sections were used for stroma evaluation. Stroma was classified as desmoplastic or fibrohyaline taking into account the density of the stromal cells, stromal edema, and collagen density in intercellular space. Necrosis was evaluated in terms of presence or absence, while two kinds of inflammatory grade including no-weak and medium-intense reaction have been established.

### 2.7. Statistical Analysis

Statistical analysis was performed using SPSS 16.0 software. Association between clinicopathological characteristics was assessed by Fischer's exact test for categorical variables and Mann-Whitney test for quantitative variables. Normality of qRT-PCR data was tested using Shapiro-Wilk test. According to data distribution, the differences in gene expression between studied groups, evaluated by qRT-PCR, were assessed by parametric tests. When two groups were considered, the comparison was made using Student's *t*-test, while for three groups we used one-way ANOVA followed by Tukey's post hoc test. A *p* value lower than 0.05 was considered significant.

## 3. Results

### 3.1. Association between Clinicopathological Parameters of the Patients and Breast Cancer Subtype

**Figure 1:**
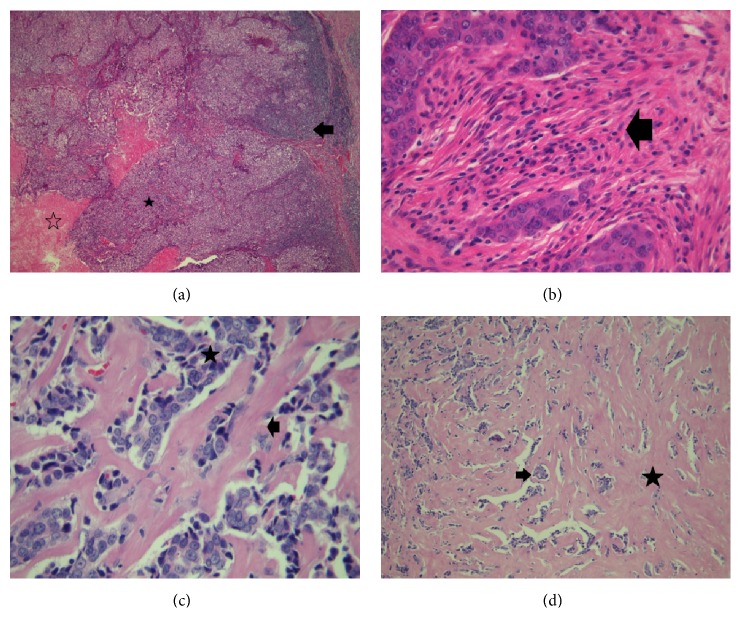
Stroma evaluation for ER−PR−HER2− (TNBC) and ER+PR+HER2− breast cancer subtypes: (a) TNBC mammary carcinoma (40x): black star, viable tumor, hollow star, necrosis, and black arrow, heavy inflammation; (b) TNBC mammary carcinoma (400x): black arrow, desmoplastic stroma with moderate inflammation; (c) ER+PR+HER2− mammary carcinoma (400x): black arrow, fibrohyaline stroma, and black star, viable tumor; (d) TNBC mammary carcinoma (40x): black arrow, viable tumor, and black star, desmoplastic stroma.

All patients included in the study were diagnosed with HER2 negative invasive ductal carcinomas. ER+PR+ and ER−PR− subtypes were approximately equally distributed among HER2− breast cancer cases (51.7% and 48.3%, resp.). ER+PR+HER2− and ER−PR−HER2− groups were comparable in age, and more than 60% of the patients had reached menopause at the date of the diagnosis. All of the TNBC cases had positive lymph nodes, and about 71% of them were Nottingham grade III. None of the patients had detectable metastasis at diagnosis. The majority of ER+PR+HER2− samples presented fibrohyaline stroma with no or weak inflammation, while in the case of ER−PR−HER2− subtype 9 out of 14 samples presented desmoplastic stroma with medium or intense inflammation (Figure 1 and Table 2).

Associations between the clinicopathological parameters and ER, PR status are presented in Table 2. TNBC subtype was significantly associated with high Nottingham grade, desmoplastic stroma, inflammation, and necrosis. No association was found for lymph nodes, tumor size, clinical stage, or menopausal status.

### 3.2. Gene Expression Profiling of Whole Blood

We have generated genome-wide transcriptional profiles of blood samples from 14 patients with triple-negative breast cancer (TNBC/ER−PR−HER2−), 15 patients with hormone-dependent breast cancer (ER+PR+HER2−), and seven healthy donors (CTR). Microarray-based gene expression analysis revealed different molecular blood signatures according to the ER/PR status. We found 371 genes with at least 1.5-fold differential changes in TNBC compared to CTR samples. Of these genes, 177 were overexpressed, and 194 were underexpressed. Following the same criteria of selection, we identified 579 genes differentially expressed in ER+PR+HER2− compared to CTR (314 upregulated and 265 downregulated genes) and 172 genes with altered expression in TNBC versus ER+PR+HER2− samples (79 upregulated and 93 downregulated genes).

**Figure 2:**
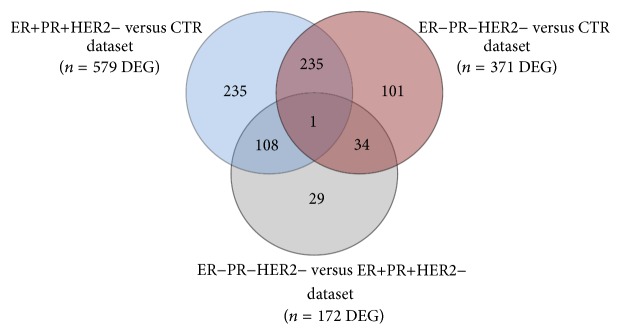
Venn diagram of genes/sequences with differential expression exceeding 1.5-fold in the compared groups. The overlap areas show unique signatures for ER+PR+HER2− (108) and ER−PR−HER2− (TNBC) (34) subtypes.

Intersecting the results for all three comparisons yielded a 108-specific signature for hormone-dependent subtype (sequences with differential expression in ER+PR+HER2− compared to CTR and TNBC but not in TNBC versus CTR) and 34-specific genes signature for TNBC subtype (sequences with differential expression in TNBC compared to CTR and ER+PR+HER2− but not in ER+PR+HER2− versus CTR) (Figure 2). The full lists of specific genes for ER−PR−HER2− and ER+PR+HER2− subtypes are presented in Additional file 1 in Supplementary Material available online at http://dx.doi.org/10.1155/2016/3239167.

**Figure 3:**
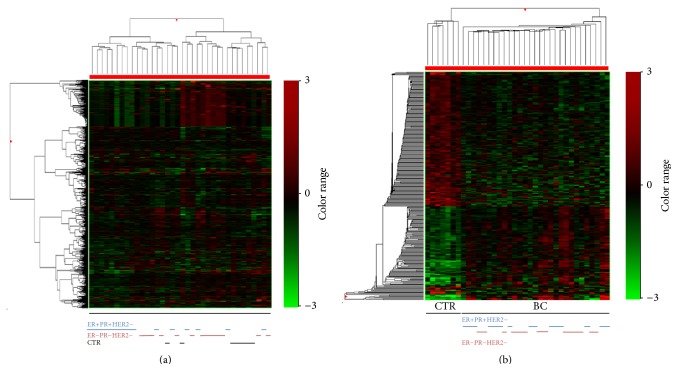
Unsupervised (a) and supervised (b) hierarchical clustering of blood samples from 29 BC and 7 CTR. The hierarchical clusters were computed using Euclidean distances and Ward method. The color indicates the level of mRNA expression: red, higher level of expression; green, lower level of expression; black, no expression change. All samples are represented by columns and genes by rows.

In order to have a global image of the transformations that occur in the blood cells of the patients with breast cancer pathology, we further considered all 29 blood samples from breast cancer patients as a whole group (BC). A disease signature with differential expression of 290 genes with greater than 1.5-fold expression changes (155 upregulated and 135 downregulated genes) was identified by microarray analysis in BC compared to CTR. The supervised hierarchical clustering of these profiles revealed two distinct clusters corresponding to CTR and BC groups. Although different molecular profiles were observed in the ER−PR−HER2− and ER+PR+HER2− subtypes, the pattern for these subgroups in the BC cluster was mixed and did not cluster according to the ER, PR status (Figure 3).

### 3.3. Assessment of Deregulated Pathways and Biological Processes

**Figure 4:**
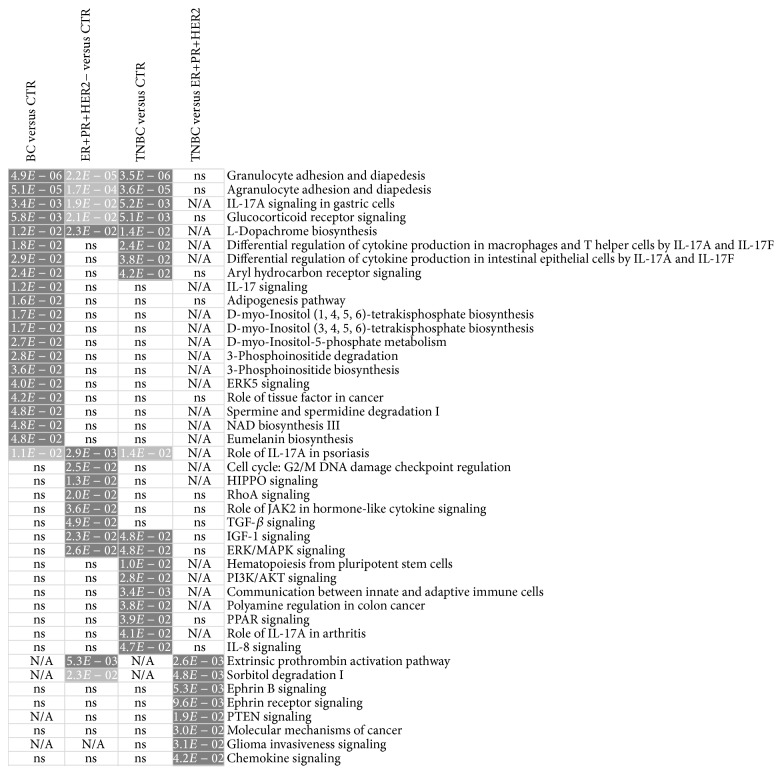
Heat map of the significant canonical pathways in the four datasets of differentially expressed genes: BC versus CTR, ER+PR+HER− versus CTR, TNBC (ER−PR−HER−) versus CTR, and TNBC versus ER+PR+HER− subgroup. The significance of the association between canonical pathways and each dataset was assessed in IPA by Fischer's exact test (*p* < 0.05). The darkest color was assigned to the smallest *p* value for a canonical pathway among all datasets, while uncolored boxes indicate the nonsignificant canonical pathways (ns) or their absence (N/A).

In order to identify the molecular pathways and biological processes affected by the transcriptional changes observed in the peripheral blood of patients with breast cancer, we analyzed our data using IPA software. We ran Core Analysis for BC versus CTR, ER−PR−HER2− versus CTR, ER+PR+HER2− versus CTR, and ER−PR−HER2− versus ER+PR+HER2− datasets. IPA analysis revealed 43 significant canonical pathways (*p* < 0.05) across the four datasets (Figure 4). The color code in the heat map of the canonical pathways is related to *p* value obtained by Fischer's exact test; the darkest color was assigned for the dataset in which the canonical pathway is the most significant. We identified 15 significant canonical pathways in the peripheral blood of ER+PR+HER2− patients, respectively, and 18 significant pathways in TNBC patients when compared to control group. Specific canonical pathways such as “communication between innate and adaptive immune cells,” “differential regulation of cytokine production in macrophages and T helper cells by IL-17A and IL-17F,” and “differential regulation of cytokine production in intestinal epithelial cells by IL-17A and IL-17F” were activated just in TNBC when compared to control group, while “chemokine signaling,” ephrin B signaling, and PTEN signaling were found only in TNBC when compared to ER+PR−HER2− subgroup.

**Figure 5:**
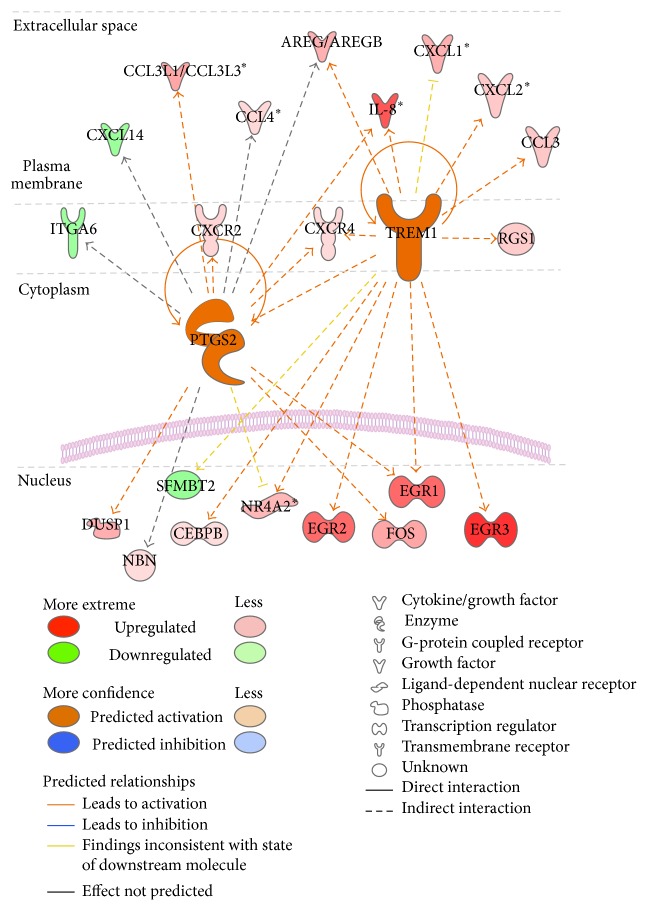
The network of TREM1 and PTGS2 (COX-2) upstream regulators and their target molecules, evaluated in the peripheral blood cells of breast cancer patients compared with healthy donors (Ingenuity Pathway Analysis).

In order to better understand the differences observed in the peripheral blood cells of patients with breast cancer we further focused on the implications of immune cells in tumor development. We identified the statistically significant biofunctions induced by innate and adaptive immune cells in breast cancer patients (Additional file 1). By evaluating the genes involved in movement, adhesion, migration, and infiltration of immune cells, we identified several upregulated proinflammatory modulators including CXCL1, CXCL2, CXCR2, CXCR4, CCL3, CCL4, CCL3L1/CCL3L3, EGR1, EGR2, EGR3, IL-8, PTSG2, PLAU, OSM, and TREM1. The assessment of key modulators, based on IPA Upstream Regulator Analysis, has highlighted two of the mentioned proinflammatory factors, PTGS2/COX-2 (*z*-score = 2.322 and overlap *p* value = 6.41*E* − 09) and TREM1 (*z*-score = 2.685 and overlap *p* value = 8.11*E* − 08), as upstream regulators when comparing all breast cancer samples with healthy donors (Table 3). The relationship between upstream regulators and their targets is presented in Figure 5. AREG/AREGB and F7 were predicted to be upstream regulators in hormone-dependent subtype, whereas only AREG/AREGB was identified as an upstream regulator in the dataset (Table 3).

### 3.4. qRT-PCR Data Validation

The common upstream regulators (PTGS2 and TREM1) and two of their common targets (IL-8 and AREG) were evaluated by qRT-PCR (Table 4). We found full concordance between qRT-PCR data and microarray results in terms of magnitude and the direction of expression changes. For PTGS2, differences in expression assessed by qRT-PCR were higher than those obtained by microarray in all comparison, while for TREM1, IL-8, and AREG the expression was comparable between microarray and qRT-PCR data.

## 4. Discussion

The interaction between tumor and host is not limited to communication with its local microenvironment but also affects distant anatomic sites and systemic immune response. Consequently, these interactions could be reflected by a tumor-related blood gene expression signature. In this study, we explored transcriptional profiles in the peripheral blood cells of HER2 negative breast cancer patients according to ER/PR status to evaluate whether there are transcriptional differences and to gain a better understanding of the changes triggered into the blood circulating cells.

Our results highlighted the implication of tumor-related inflammation as well as the immune response in all blood samples from breast cancer patients, with an enrichment of these processes in the TNBC subtype. Tumor microenvironment (TME), a springboard for tumor growth, represents a complex cooperation between tumor, stroma, and blood cells [16, 17]. In the new conceptual rationale, Hanahan and Coussens [18] described the TME including tumor-promoting inflammation as a new hallmark of cancer, with immune cells being recognized to facilitate the cancer progression. Inflammatory cells and inflammatory mediators including chemokines, cytokines, and prostaglandins were identified in the TME of most tumors, including breast cancers [19, 20].

Increasing evidence suggests that, besides tumor cells, cancer-associated fibroblasts (CAFs), tumor associated macrophages (TAMs), and tumor-associated neutrophils (TANs) are involved in the production of proinflammatory chemokines and cytokines. Cancer-associated fibroblasts (CAFs), an activated population of stromal fibroblasts with a role in tumor development, represent the most important stromal cell type of mediators of tumor-promoting inflammation [21]. Previous studies have shown that CAFs production of CXCL1, CXCL2, PTGS2/COX-2, and IL-6 in breast cancer can modulate the functions of immune cells in TME [22, 23]. Our data showed overexpression of proinflammatory factors including CXCL1, CXCL2, CXCR4, CCL3, CCL4, IL-8, and PTGS2/COX-2 in the peripheral blood of patients with breast cancer both when considering the subtypes individually and when considering all breast cancer samples as a group. Furthermore, we found that PTGS2/COX-2's targets were highly enriched in BC versus CTR samples (overlap *p* value = 6.41*E* − 09) (Table 3). Recent studies indicated that tumor expression of COX-2 can lead to epithelial to mesenchymal transition in breast tumor cells [24], and the pharmacological inhibition of COX-2 reduces breast tumor development [25, 26]. Although PTGS2/COX-2 was identified as a possible predictive marker of micrometastasis of breast cancer in the bone marrow [27], currently there are no studies to show its overexpression in the blood of patients with breast cancer. In preneoplastic lesions, proinflammatory cytokines and chemokines secreted by CAFs are involved in complex regulatory signals promoting macrophage recruitment, angiogenesis, and tumor growth [28].

On the other hand, circulating monocytes represent the source of TAMs that are selectively attracted in TME by tumor-derived attractants such as chemokines and cytokines [29]. Substantial evidence suggests that TAMs accumulate preferentially in hypoxic regions of tumors, leading to overexpression of COX-2, VEGF, IL-8, CXCL12, or CXCR4 [30]. COX-2 overexpression in turn leads to increased production of proangiogenic factors such as VEGF, IL-8, and CXCL1 with a role in vascular channel formation [31, 32]. Our data showed a significant activation of “IL-8 signaling” pathway in the blood cells of TNBC patients correlated with increased inflammation and necrosis in primary tumors.

It is known that IL-8 released by tumor cells represents a chemoattractant for neutrophils to TME [33]. Additionally, in a similar manner to that in wounds, neutrophils secrete cytokines including IL-6, IL-8, TNF-*α*, and GM-CSF, enhancing angiogenesis and tumor progression. Most of TNBC are highly proliferative tumors and have poor developed vascular networks that generate susceptibility to hypoxia and implicit necrosis. Tumor necrosis has been associated with a poor outcome in breast carcinoma [34] and usually generates cytokine-like IL-1 and HMGB1 with a role of promoting inflammatory response and neoangiogenesis [35]. Our data highlight a high cellular density of inflammatory and fibroblastic cells in stroma of TNBC subtypes. In a recent study, Pierobon et al. [36] demonstrated that, in hypoxic conditions, TREM1, a transmembrane receptor expressed in myeloid cells, is involved in the inflammatory responses mediated by neutrophils and monocytes. TREM1 is considered to amplify inflammation by triggering the secretion of some important inflammatory factors including TNF-*α*, IL-6, CXCL8, CCL4, and CCL5 [37]. In our study, TREM1 was revealed as an upstream regulator in the peripheral blood of BC patients (*z*-score = 2.685; overlap *p* value = 8.11*E* − 09). Our data indicates that TREM1 activates important proinflammatory factors including IL-8, CXCL1, CXCL2, CXCL3, AREG, and transcription regulators such as EGR1, EGR2, and EGR3, suggesting enhancing in differentiation and mitogenesis. Balzarolo et al. [38] showed that EGR1, when activated, acts as a brake on TNF-related apoptosis-inducing ligand (TRAIL) expression in NK cells. The role of the soluble protein TREM1 as possible prognostic marker to detect lung metastasis was previously reported [39]; nevertheless no study has investigated the role of TREM1 in the peripheral blood of breast cancer patients.

Cross talk between innate and adaptive immune systems has already been demonstrated to have profound effects on cancer development, including breast carcinogenesis [40, 41]. The immune system balance was also confirmed by our study. Our data revealed that “differential regulation of cytokine production in macrophages and T helper cells by IL-17A and IL-17F” canonical pathway was activated just in the blood of TNBC patients but not in ER+PR+HER2− patients when compared to CTR group (Table 3). The role of IL-17A and IL-17F was previously related to neutrophils recruitment during inflammation. These molecules activate fibroblasts from both innate and epithelial cells from TME to produce proinflammatory cytokines and chemokines [42]. Furthermore, an enrichment of “chemokine signaling” was observed in the blood of patients with TNBC subtype compared to hormone-dependent subtype.

Our results highlighted activation of processes such as mobilization, migration, infiltration, and accumulation of leucocytes in the blood of the patients with breast cancer (Additional file 1). The set of cytokines, chemokines, and biomediators identified as upregulated in peripheral blood cells of breast cancer patients have been shown to stimulate innate peripheral blood immune cells such as phagocytes and granulocytes to migrate and infiltrate to TME. It is known that leucocytes represent crucial regulators of cancer development by altering local homeostasis and declining immune balance between antitumor responses and oncogenic pathways [43]. Our data suggest a more extensive immune response in patients with TNBC compared to that with ER+PR+HER2− subtype by a significant accumulation, trafficking/migration, and adhesion of phagocytes and granulocytes. Processes such as migration of inflammatory leucocytes were also revealed as more significantly upregulated in TNBC versus ER+PR+HER2− samples. Furthermore, human breast carcinoma that contains infiltrates of innate-immune cell types such as macrophages was associated with increased angiogenesis and unfavorable clinical prognosis [44]. An increased process related to the accumulation of macrophages (*p* = 0.0059) (Additional file 1) and more pathways involved in modulation of angiogenesis (Figure 3) were observed in TNBC versus hormone-dependent subtype.

We noticed more functions related to proliferation, differentiation, and migration of peripheral blood lymphocyte in BC patients, with more intense T cells activities, especially in the TNBC subtype (Additional file 1). It is widely known that T cells are the most potent cells of the immune system. In TNBC subtype, we observed a shifting of immune responses toward accumulation, transmigration, and conversion of naive T lymphocyte, balancing innate, and adaptive immunity but accompanied by inhibition of B lymphocytes maturation.

The interactions between TME and cancer cells represent intrinsic features of breast cancer subtypes. In a recent study, Camp et al. [45] identified specific microenvironment features when comparing basal-like with luminal breast cancer subtypes. They observed that basal-like cells respond to stromal interactions by increasing migration, including important proinflammatory mediators such as IL-6, IL-8, CXCL1, and oncostatin M (OSM). Our data also indicate the presence of these molecules in the peripheral blood cells of patients with breast cancer regardless of subtype. Furthermore, validation of our results on larger cohorts could contribute to a better understanding of the role of immune system in HER2− breast cancer subtypes, allowing new immunotherapeutic approaches.

In conclusion, our data show distinct molecular signatures in the blood of HER2 negative breast cancer patients according to ER/PR status. We noticed a significant enrichment of altered systemic immune-related pathways in the blood of TNBC patients correlated with an increased inflammation and necrosis in primary tumors suggesting that immunotherapy could possibly be synergistic to the chemotherapy to improve the clinical outcome of these patients.

## Supplementary Material

The Additional file 1 contains the full lists of specific genes/sequences with their fold change and description for ER−PR−HER2− and ER+PR+HER2− subtypes, as well as the statistically significant biofunctions for innate and adaptive immune cells, focused on the implications of immune cells in tumor development.

## Figures and Tables

**Table 1:** Baseline clinical and histological characteristics of the HER2− patients.

Number	Age	Clinical stage	TNM staging	Nottingham score	Menopause age	ER/PR status
1	58	II B	T2N1M0	II	#	ER−/PR−
2	53	III A	T2N2M0	II	50	ER−/PR−
3	40	III B	T4bN2M0	II	39	ER−/PR−
4	45	III A	T3N1M0	III	#	ER−/PR−
5^*∗*^	48	II B (R)/I A (L)	T2N1M0(R)/T1N0M0(L)	III	32	ER−/PR−
6	49	II B	T2N1M0	III	#	ER−/PR−
7	50	III B	T4bN1M0	II	#	ER−/PR−
8^*∗*^	55	III B (R)/I B (L)	T4bN2M0(R)/T1N0M0 (L)	III	51	ER−/PR−
9	56	II B	T2N1M0	III	N/A	ER−/PR−
10	60	II A	T1N1MO	III	45	ER−/PR−
11	35	II B	T2N1M0	III	#	ER−/PR−
12	53	III A	T2N2M0	III	50	ER−/PR−
13	40	II B	T2N1M0	III	#	ER−/PR−
14	74	III B	T4bN2M0	III	48	ER−/PR−
15	50	II B	T2N1M0	II	50	ER+/PR+
16	45	II B	T2N1M0	I	45	ER+/PR+
17	52	III A	T2N2M0	III	N/A	ER+/PR+
18	54	III A	T2N2M0	II	#	ER+/PR+
19	65	III B	T4bN2M0	II	47	ER+/PR+
20	50	I	T1cN0M0	III	45	ER+/PR+
21	62	III B	T4bN2M0	II	54	ER+/PR+
22	52	III B	T4bN2M0	III	50	ER+/PR+
23	62	III A	T3N1M0	II	52	ER+/PR+
24	68	II B	T3N0M0	I	50	ER+/PR+
25	49	III A	T3N1M0	I	#	ER+/PR+
26	43	III A	T3N1M0	I	44	ER+/PR+
27	63	III B	T4aN0M0	II	40	ER+/PR+
28	52	II B	T2N1M0	II	52	ER+/PR+
29	48	II A	T2N0M0	II	#	ER+/PR+

^*∗*^Patients with bilateral breast cancer: R, right breast tumor; L, left breast tumor.

#: the patient has been diagnosed before reaching menopause; N/A: missing data.

**Table 2:** Association between ER, PR status and clinicopathological parameters.

Characteristics	Number of patients (%)	ER+PR+HER2−	ER−PR−HER2−	*p* value
Study population	**29 (100%)**	**15 (51.7%)**	**14 (48.3%)**	
Median age (years)		52	51.5	0.41
Menopausal status				
Pre	9 (31%)	11	7	0.24
Post	18 (62.1%)	3	6	
N/A	2 (6.9%)	1	1	
Clinical stage^§^				
I-II	13 (44.8%)	6	7	0.71
III	16 (55.2%)	9	7	
Tumor size^§^				
T1-T2	16 (55.2%)	7	9	0.46
T3-T4	13 (44.8%)	8	5	
Lymph nodes^§^				
N0	4 (13.8%)	4	0	—
N1	15 (51.7%)	6	9	
N2	10 (34.5%)	5	5	
Nottingham grading				
I-II	16 (55.2%)	12	4	**0.009**
III	13 (44.8%)	3	10	
Stroma				
Fibrohyaline	17 (58.6%)	12	5	**0.025**
Desmoplastic	12 (41.4%)	3	9	
Inflammation				
No-weak	18 (62.1%)	13	5	**0.008**
Medium-intense	11 (37.9%)	2	9	
Necrosis				
Absent	21 (72.4%)	15	6	**0.0007**
Present	8 (27.6%)	0	8	

^§^Two patients with bilateral cancer. The higher value for clinical stage, tumor size, and nodes was considered.

**Table 3:** Upstream regulators predicted by IPA in the peripheral blood cells of breast cancer patients compared with healthy donors.

Upstream regulator	Fold change	Molecule type	Predicted activation state	*z*-score	*p* value of overlap	Target molecules in dataset
*BC versus CTR*						
PTGS2	5.764	Enzyme	Activated	2.322	6.41*E* − 09	AREG/AREGB, CCL3L1/CCL3L3, CCL4, CXCL14, CXCR2, CXCR4, DUSP1, EGR1, FOS, IL-8, ITGA6, NBN, NR4A2, PTGS2
TREM1	2.051	Transmembrane receptor	Activated	2.685	8.11*E* − 08	AREG/AREGB, CCL3, CEBPB, CXCL1, CXCL2, CXCR4, EGR1, EGR2, EGR3, IL-8, NR4A2, PTGS2, RGS1, SFMBT2
*ER+PR+HER*−* versus CTR*						
F7	−2.303	Peptidase	Activated	2.736	1.03*E* − 07	CXCL2, EGR1, FOS, IER2, IL-8, JAG1, KLF5, ZFP36
AREG	4.422	Growth factor	Activated	2.395	2.45*E* − 06	AREG/AREGB, CXCR4, EGR1, FOS, NPPC, PTGS2
*ER*−*PR*−*HER2*−* versus CTR*						
AREG	2.803	Growth factor	Activated	2.407	1.12*E* − 06	AREG/AREGB, CXCR4, EGR1, FOS, PLAU, PTGS2

**Table 4:** Relative expression of PTSG2, TREM1, IL-8, and AGREG assessed by qRT-PCR in BC, ER−PR−HER2−, and ER+PR+HER2− samples. Statistical significance was assessed using one-way ANOVA followed by Tukey's post hoc test.

	BC versus CTR		ER−PR−HER2− versus CTR		ER+PR+HER2− versus CTR	
	FR	*p* value	FR	*p* value	FR	*p* value
PTGS2	11.36	<0.0001	11.21	<0.0001	11.50	<0.0001
TREM 1	1.88	0.005	1.93	0.013	1.83	0.043
IL-8	6.99	<0.0001	5.98	<0.0001	7.94	<0.0001
AREG	4.16	<0.0001	4.07	<0.0001	4.26	<0.0001

## References

[1] Ferlay J., Soerjomataram I., Dikshit R. (2015). Cancer incidence and mortality worldwide: sources, methods and major patterns in GLOBOCAN 2012. *International Journal of Cancer*.

[2] Perou C. M., Sørile T., Eisen M. B. (2000). Molecular portraits of human breast tumours. *Nature*.

[3] Sørlie T., Perou C. M., Tibshirani R. (2001). Gene expression patterns of breast carcinomas distinguish tumor subclasses with clinical implications. *Proceedings of the National Academy of Sciences of the United States of America*.

[4] Sørlie T., Tibshirani R., Parker J. (2003). Repeated observation of breast tumor subtypes in independent gene expression data sets. *Proceedings of the National Academy of Sciences of the United States of America*.

[5] Curtis C., Shah S. P., Chin S.-F. (2012). The genomic and transcriptomic architecture of 2,000 breast tumours reveals novel subgroups. *Nature*.

[6] Rivenbark A. G., O'Connor S. M., Coleman W. B. (2013). Molecular and cellular heterogeneity in breast cancer: challenges for personalized medicine. *American Journal of Pathology*.

[7] Dent S., Oyan B., Honig A., Mano M., Howell S. (2013). HER2-targeted therapy in breast cancer: a systematic review of neoadjuvant trials. *Cancer Treatment Reviews*.

[8] Alizadeh A. A., Elsen M. B., Davis R. E. (2000). Distinct types of diffuse large B-cell lymphoma identified by gene expression profiling. *Nature*.

[9] Hong J., Zang Y. C. Q., Hutton G., Rivera V. M., Zhang J. Z. (2004). Gene expression profiling of relevant biomarkers for treatment evaluation in multiple sclerosis. *Journal of Neuroimmunology*.

[10] Olsen N. J., Moore J. H., Aune T. M. (2004). Gene expression signatures for autoimmune disease in peripheral blood mononuclear cells. *Arthritis Research and Therapy*.

[11] Sharma P., Sahni N. S., Tibshirani R. (2005). Early detection of breast cancer based on gene-expression patterns in peripheral blood cells. *Breast Cancer Research*.

[12] Aarøe J., Lindahl T., Dumeaux V. (2010). Gene expression profiling of peripheral blood cells for early detection of breast cancer. *Breast Cancer Research*.

[13] Smirnov D. A., Foulk B. W., Doyle G. V., Connelly M. C., Terstappen L. W. M. M., O'Hara S. M. (2006). Global gene expression profiling of circulating endothelial cells in patients with metastatic carcinomas. *Cancer Research*.

[14] Zuckerman N. S., Yu H., Simons D. L. (2013). Altered local and systemic immune profiles underlie lymph node metastasis in breast cancer patients. *International Journal of Cancer*.

[15] Whiteside T. L. (2014). Regulatory T cell subsets in human cancer: are they regulating for or against tumor progression?. *Cancer Immunology, Immunotherapy*.

[16] Jaillon S., Galdiero M. R., Del Prete D., Cassatella M. A., Garlanda C., Mantovani A. (2013). Neutrophils in innate and adaptive immunity. *Seminars in Immunopathology*.

[17] Mantovani A., Cassatella M. A., Costantini C., Jaillon S. (2011). Neutrophils in the activation and regulation of innate and adaptive immunity. *Nature Reviews Immunology*.

[18] Hanahan D., Coussens L. M. (2012). Accessories to the crime: functions of cells recruited to the tumor microenvironment. *Cancer Cell*.

[19] Tan W., Zhang W., Strasner A. (2011). Tumour-infiltrating regulatory T cells stimulate mammary cancer metastasis through RANKL-RANK signalling. *Nature*.

[20] Lewis C. E., Hughes R. (2007). Inflammation and breast cancer. Microenvironmental factors regulating macrophage function in breast tumours: hypoxia and angiopoietin-2. *Breast Cancer Research*.

[21] Servais C., Erez N. (2013). From sentinel cells to inflammatory culprits: cancer-associated fibroblasts in tumour-related inflammation. *Journal of Pathology*.

[22] Mantovani A., Allavena P., Sica A., Balkwill F. (2008). Cancer-related inflammation. *Nature*.

[23] Erez N., Glanz S., Raz Y., Avivi C., Barshack I. (2013). Cancer associated fibroblasts express pro-inflammatory factors in human breast and ovarian tumors. *Biochemical and Biophysical Research Communications*.

[24] Bocca C., Ievolella M., Autelli R. (2014). Expression of Cox-2 in human breast cancer cells as a critical determinant of epithelial-to-mesenchymal transition and invasiveness. *Expert Opinion on Therapeutic Targets*.

[25] Basu G. D., Pathangey L. B., Tinder T. L., LaGioia M., Gendler S. J., Mukherjee P. (2004). Cyclooxygenase-2 inhibitor induces apoptosis in breast cancer cells in an in vivo model of spontaneous metastatic breast cancer. *Molecular Cancer Research*.

[26] Na Y.-R., Yoon Y.-N., Son D.-I., Seok S.-H. (2013). Cyclooxygenase-2 inhibition blocks M2 macrophage differentiation and suppresses metastasis in murine breast cancer model. *PLoS ONE*.

[27] Zhu L., Loo W. T. Y., Cheng C. W. N., Chow L. W. C. (2006). Possible predictive markers related to micro-metastasis in breast cancer patients. *Oncology Reports*.

[28] Erez N., Truitt M., Olson P., Hanahan D. (2010). Cancer-associated fibroblasts are activated in incipient neoplasia to orchestrate tumor-promoting inflammation in an NF-kappaB-dependent manner. *Cancer Cell*.

[29] Sica A., Allavena P., Mantovani A. (2008). Cancer related inflammation: the macrophage connection. *Cancer Letters*.

[30] Murdoch C., Lewis C. E. (2005). Macrophage migration and gene expression in response to tumor hypoxia. *International Journal of Cancer*.

[31] Robertson F. M., Mallery S. R., Bergdall-Costell V. K. (2007). Cyclooxygenase-2 directly induces MCF-7 breast tumor cells to develop into exponentially growing, highly angiogenic and regionally invasive human ductal carcinoma xenografts. *Anticancer Research*.

[32] Basu G. D., Liang W. S., Stephan D. A. (2006). A novel role for cyclooxygenase-2 in regulating vascular channel formation by human breast cancer cells. *Breast Cancer Research*.

[33] Houghton A. M. (2010). The paradox of tumor-associated neutrophils: fueling tumor growth with cytotoxic substances. *Cell Cycle*.

[34] Gilchrist K. W., Gray R., Fowble B., Tormey D. C., Taylor S. G. (1993). Tumor necrosis is a prognostic predictor for early recurrence and death in lymph node-positive breast cancer: a 10-year follow-up study of 728 eastern cooperative oncology group patients. *Journal of Clinical Oncology*.

[35] Vakkila J., Lotze M. T. (2004). Inflammation and necrosis promote tumour growth. *Nature Reviews Immunology*.

[36] Pierobon D., Bosco M. C., Blengio F. (2013). Chronic hypoxia reprograms human immature dendritic cells by inducing a proinflammatory phenotype and TREM-1 expression. *European Journal of Immunology*.

[37] Bosco M. C., Pierobon D., Blengio F. (2011). Hypoxia modulates the gene expression profile of immunoregulatory receptors in human mature dendritic cells: identification of TREM-1 as a novel hypoxic marker in vitro and in vivo. *Blood*.

[38] Balzarolo M., Watzl C., Medema J. P., Wolkers M. C. (2013). NAB2 and EGR-1 exert opposite roles in regulating TRAIL expression in human Natural Killer cells. *Immunology Letters*.

[39] Karapanagiotou E. M., Pelekanou E., Charpidou A. (2008). Soluble triggering receptor expressed on myeloid cells-1 (sTREM-1) detection in cancer patients: a prognostic marker for lung metastases from solid malignancies. *Anticancer Research*.

[40] Disis M. L. (2010). Immune regulation of cancer. *Journal of Clinical Oncology*.

[41] DeNardo D. G., Coussens L. M. (2007). Inflammation and breast cancer. Balancing immune response: crosstalk between adaptive and innate immune cells during breast cancer progression. *Breast Cancer Research*.

[42] Reynolds J. M., Angkasekwinai P., Dong C. (2010). IL-17 Family member cytokines: regulation and function in innate immunity. *Cytokine and Growth Factor Reviews*.

[43] de Visser K. E., Eichten A., Coussens L. M. (2006). Paradoxical roles of the immune system during cancer development. *Nature Reviews Cancer*.

[44] Tsutsui S., Yasuda K., Suzuki K., Tahara K., Higashi H., Era S. (2005). Macrophage infiltration and its prognostic implications in breast cancer: the relationship with VEGF expression and microvessel density. *Oncology Reports*.

[45] Camp J. T., Elloumi F., Roman-Perez E. (2011). Interactions with fibroblasts are distinct in basal-like and luminal breast cancers. *Molecular Cancer Research*.

